# Pre-exposure prophylaxis use among HIV serodiscordant couples: a qualitative study in Mozambique

**DOI:** 10.1080/16549716.2021.1940764

**Published:** 2021-07-06

**Authors:** Daniel E. Sack, Caroline De Schacht, Paula Paulo, Erin Graves, Almiro M. Emílio, Ariano Matino, Carlota L. Fonseca, Arifo U. Aboobacar, Sara Van Rompaey, Carolyn M. Audet

**Affiliations:** a Vanderbilt Institute for Global Health, Vanderbilt University Medical Center, Nashville, TN, USA; b Friends in Global Health, Maputo, Mozambique; c Friends in Global Health, Quelimane, Mozambique

**Keywords:** PrEP, rural Africa, discordant couples, HIV/AIDS, Mozambique

## Abstract

**Background:**

Pre-exposure prophylaxis (PrEP) has the potential to reduce HIV transmission and stem the HIV epidemic. Unfortunately, PrEP uptake in rural sub-Saharan Africa has been slow and medication adherence has been suboptimal.

**Objective:**

To explore the perspectives, attitudes, and experiences of HIV serodiscordant partners taking PrEP and develop a messaging campaign to improve PrEP uptake in rural Mozambique to reduce HIV transmission among serodiscordant partners.

**Methods:**

In this qualitative study, we interviewed 20 people in serodiscordant relationships using PrEP at a rural health center in Zambézia province, Mozambique and employed inductive and deductive coding to elicit their perspectives, attitudes, and experiences related to learning their partner’s HIV status, barriers to PrEP uptake, obstacles to PrEP adherence, and decisions to disclose their PrEP use with family and friends using thematic analysis.

**Results:**

Our analysis generated nine themes across various levels of the socioecological model. Participants reported a strong desire to stay in the discordant relationship and highlighted the importance of working together to ensure PrEP and antiretroviral therapy adherence, with the majority skeptical that adherence could be achieved without both partners’ support (individual and interpersonal). Although most participants were reticent about sharing their serodiscordant status with family and friends (individual and interpersonal), those who did found their family and friends supportive (interpersonal). Participants suggested increasing community health agent availability to help people navigate HIV prevention and treatment (organizational). We then created three oral stories, using themes from the interviews, with examples from various levels of the socioecological model that will be used to generate support for PrEP use among community members.

**Conclusions:**

Our findings informed oral template stories that will be used to emphasize how couples can work together to improve PrEP uptake and reduce incident HIV infections in serodiscordant couples elsewhere in rural Mozambique.

## Background

Pre-exposure prophylaxis (PrEP) uptake and retention is key to reducing HIV transmission and the global burden of HIV [1–5]. Low medication adherence in initial clinical trials made it difficult to assess PrEP’s ability to prevent HIV transmission to women in serodiscordant relationships living in sub-Saharan Africa [6–11]. Although HIV transmission within serodiscordant couples, which accounts for 5–53% of HIV transmission in the region, occurs at a rate of approximately 11.1 per 100 person-years in sub-Saharan Africa, people in serodiscordant relationships report several barriers to PrEP uptake and adherence [12–15]. These obstacles include discomfort discussing PrEP/ART, a lack of knowledge about PrEP, and confusion navigating sexual encounters (e.g. condom use with primary partners) [14,15]. Serodiscordant couples also report that their fear of contracting HIV and stigma related to leaving their partner makes it difficult to stay in the relationship (particularly for women with a seropositive partner) [14,15].

PrEP implementation challenges in sub-Saharan Africa have been attributed to medication regulatory requirements, cost effectiveness concerns, a lack of health system capacity, HIV stigma, and medication adherence and efficacy concerns [16,17]. In 2018, PrEP became available for people in serodiscordant relationships at district level health facilities in Zambézia province, Mozambique [18]. Zambézia is a rural, north-central province with approximately 5.1 million people, with some of the lowest health and development indicators in Mozambique [19–21]. Its HIV prevalence, approximately 15%, is among the highest in the country, 30% of people living with HIV are on ART, and 49.5% of people on ART are virally suppressed (23.1% of all people living with HIV) [19]. Although healthcare providers were aware of clinical guidelines for PrEP management, among the few thousand individuals who initiated PrEP, about half stopped taking it after 2–6 months and misunderstandings about and stigma related to PrEP persisted among those at risk for HIV during the initial rollout [18].

In this qualitative study we assessed attitudes and perceptions about PrEP use in people in serodiscordant relationships who had initiated PrEP at a district health facility in Zambézia. After analyzing participant responses, we developed three oral stories designed to educate, empower, and normalize PrEP use. These stories will be presented to discordant couples to try improve PrEP uptake and reduce incident HIV infections in rural Mozambique.

## Methods

### Study design

This cross-sectional, qualitative study employed thematic analysis to analyze in-depth interviews and develop oral stories intended to improve PrEP uptake in rural Mozambique.

### Participant sampling

We approached all adults (≥18 years old) who reported they were HIV-negative, in a serodiscordant relationship, and had ever taken or were currently taking PrEP between 28 November 2019 to 24 January 2020 at a primary care clinic in Namacurra, Mozambique due to the availability of study personnel at the clinic. Nurses at the clinic informed patients using PrEP of the study and referred those who expressed interest to a research assistant to assess eligibility. We recruited participants until we reached data saturation [22]. Of the 22 patients referred to study personnel, 20 were eligible and agreed to participate.

### Data collection

Between November 2019 and January 2020, we conducted 20 semi-structured in-depth interviews (Table 1). Fieldworkers had more than ten years of experience conducting qualitative interviews in Zambézia Province and received a five-day refresher training course in qualitative interview methods, including time to craft and improve our interview guide, originally developed based on our experience working in the area, before data collection began (Table 1). Interview training included sessions on developing rapport (e.g. introducing themselves, using colloquial phrasing, establishing eye contact, asking follow-up questions, and interviewing in a private location) and establishing trustworthiness (e.g. explaining the study’s purpose, describing data storage and access systems to ensure privacy, responding to the participants’ questions). Interviews included member checking, where fieldworkers summarized important details to ensure they accurately understood participants’ opinions and experiences. Fieldworkers also employed an iterative approach to add additional questions/probes based on previous respondents’ feedback. Interviews lasted 20–50 minutes, were conducted one-on-one in Portuguese (8) or Echuabo (12), and took place at the clinic or another meeting place at the convenience of the interviewee. Interviews were audio-recorded and all interviews, with one exception, were transcribed, translated into Portuguese if conducted in Echuabo, and then reverified. We excluded one interview from our analysis because we could not verify the quality of the transcription after the audio was accidently deleted after the first transcription. Two researchers translated the 19 remaining interviews from Portuguese into English for analysis.

**Table 1.** Qualitative interview guide

1.	How did you react to finding out you were HIV-negative and your partner was HIV-positive? (*probe: did you consider leaving the relationship? What emotions/thoughts came up for you, or both of you together?*)	
2.	Did you tell any other people about the results you learned? If so, what were the reactions of people you told?	
3.	Tell me what you were told about PrEP from the nurse/health care provider? What were the concerns you had about it? What benefits did you see in taking it? What influenced your decision to start taking it?	
4.	Did you tell your partner that you were taking PrEP? What was his/her reaction? How did their reaction make you feel?	
5.	How do you or did you feel about taking the medication? Do you or did you take the medication regularly as it was prescribed? If you are taking it intermittently, what information would help you be more adherent to your medication?	
6.	Has anyone in your family known that you are or were taking PrEP medication? How have they reacted to this? If you have stopped taking the medication, do you think having a more supportive family member(s) would have helped you be more adherent to your medication (i.e. stay on PrEP medication longer)?	
7.	People often learn more from stories than from listening to facts. We were thinking about offering people information about PrEP through storytelling. Imagine these three different situations:	
	a.	*A couple who struggles to overcome the difficulties associated with having a different HIV status compared to their partner. Specifically, the woman accuses the man of cheating on her and she is frustrated that she now has to take PrEP medication. Mostly she is scared that she will get HIV.*
		What is the best way for the couple to stay together and be supportive of each other’s health?
	b.	*A woman has an HIV-positive partner who refuses to take ART medication and doesn’t support her taking PrEP. She chooses to take her medication in secret and attend clinic visits in secret as well.*
		Do you think a woman could manage this? What would be the best way to support her? (ex. other family? Friends? Health care workers? Educate her partner?)
	c.	*A serodiscordant couple is very supportive of each other. They help each other remember to take their medications and help out with chores when one isn’t feeling well. The problem is that their extended family is not supportive of them staying together or the woman taking PrEP. They are rude to both the partners and spread rumors about them.*
		How can a couple best manage this type of situation? What would be the best way to support them?

Italicized text indicates question probes or scenarios that precede questions

PrEP: Pre-Exposure Prophylaxis

### Data analysis

Researchers DES and CMA reviewed the English transcripts and conducted thematic analysis with MAXQDA20© software. Researchers DES and CMA first familiarized themselves with the data by doing three read throughs of each interview and taking notes about initial comments relevant to PrEP uptake. Subsequently, we developed the initial codes deductively, guided by the socioecological model and previous research about barriers to and facilitators of PrEP adherence [17,23,24]. The socioecological model classifies themes or interventions as impacting the individual, interpersonal, organizational, or community level of influence/change and provides a useful framework for thinking about interventions that aim to prevent HIV transmission given the complex sociocultural drivers of the HIV epidemic [23–25]. In vivo (inductive) codes were generated when unique barriers to and facilitators of PrEP adherence were identified on repeated reading. We identified patterns among our codes, interpreting their broader significance employing the individual, interpersonal, organizational/clinic, and community levels of the socioecological model [23]. Over five meetings, DES and CMA met to develop, define, and compare their coding, after which there was complete agreement in the application of deductive and inductive codes and sub-codes. Codes where then assigned to overarching themes [26].

### Oral story development

We subsequently incorporated common themes relevant to support PrEP uptake and adherence into three oral stories that will be used to educate community members about PrEP, generate family member support for the discordant couple, and de-stigmatize PrEP use. The stories integrate into Mozambique’s rich history of oral storytelling [27–29]. We used the socioecological model and our understanding of how it’s used in the HIV prevention literature to guide how we structured our stories [23,25]. While we focused on including individual and interpersonal factors as facilitators of PrEP uptake within serodiscordant couples, we also provided examples of how couples could leverage organizational and community-level support [23].

## Results

**Figure 1.**
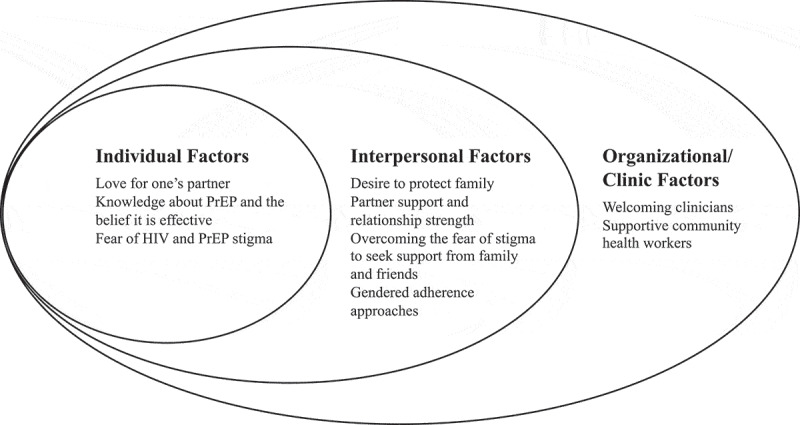
Socioecological factors influencing pre-exposure prophylaxis use among couples in HIV discordant relationships in rural Mozambique

Among the 19 participants (median age 35, interquartile range 26.5–37.5) included in analysis, 11 were female (median age 32, interquartile range 25.5–36) and 8 were male (median age 36, interquartile range 31–38). We identified 26 sub-codes across interviews, which fit into nine themes organized within levels of the socioecological model (Figure 1). These included three individual level themes – 1) love for one’s partner, 2) knowledge that PrEP exists and is effective, 3) fear of HIV and PrEP stigma – four interpersonal level themes – 4) desire to protect family, 5) partner support and relationship strength, 6) support from family and friends, 7) gendered adherence approaches – and two organizational level themes – 8) welcoming clinicians, and 9) supportive community health workers. We have mostly framed our themes as positive because we are developing stories intended to present strategies and examples of effective PrEP use. Love for one’s partner, for example, highlights how a strong relationship increases PrEP uptake; however, a lack of love within a relationship signals a barrier to PrEP uptake. Other themes, however, contain both positive and negative influences on behavior (e.g. gendered uptake and adherence approaches) or are entirely negative (e.g. fear of PrEP and HIV stigma). The themes highlight how participants reacted to their partner’s HIV diagnosis, how couples worked together to adhere to their medicines, with whom and why they did or did not discuss PrEP, and participant suggestions for future interventions. Using the socioecological model, we then describe the key characteristics of the three oral stories we created based on participant responses (Table 2).

**Table 2.** Oral story framework

	Situation	Facilitators of PrEP Use	Barriers to PrEP Use	Strategies to Overcome Barriers
*1. A Positive Story*	Partners are screened for HIV at a postnatal visit for their fourth child and the male partner tests positive while his female partner tests negative	The couple loves each other They want to be there for their children and new baby The couple listens to each other’s fears They encourage each other to adhere to their medications	Lack of knowledge about the purpose of PrEP Fear about how others in their community may react to learning that the male partner has HIV	They brought their questions to a health counsellor at the clinic They decide to keep their serodiscordant status to themselves to avoid stigma from their community
*2. An Unsupportive Family*	Partners are screened for HIV at the first prenatal care visit for their first child and the male partner tests positive while his female partner tests negative	The couple was tested together and received counselling together The couple want to keep themselves and their child healthy	The female partner is worried that her partner’s diagnosis means he was unfaithful They are scared to tell their family, as they are concerned that they will be judged for staying together Their family finds their medications and is upset and makes accusations	The clinic health counsellor explains the different ways that HIV can spread The couple engaged with a Peer Educator, who helped facilitate a conversation with the couple’s family, who then became supportive
*3. A ‘Difficult’ Partner*	Partners are screened for HIV at a prenatal care visit for their third child and the male partner tests positive while his female partner tests negative	The couple knew about PrEP from a neighbour and were initially interested	The male partner is angry about his diagnosis and the side effects from his ART and does not want to take them	The female partner requests assistance from the Peer Educator to help her male partner understand how taking his medications will protect his family and educate him about how long to expect the medication side effects to last The partners work together to remind each other to take their medications

PrEP: Pre-Exposure Prophylaxis

ART: Antiretroviral Therapy

### Individual factors influencing PrEP uptake and adherence

#### Love for one’s partner

Although participants were largely motivated by the concern for their own health and that of their children, many noted that their love and affection for their partner could help overcome challenges accompanying living in a discordant relationship. Four participants spoke about love as their motivation to remain adherent to medication. One woman explained:

*… where there is love, no one can destroy. It is up to these two if in fact they have to reach this point of consensus because they talked, they saw that it seems that life is still going on, so we will continue, we will be doing this treatment. No listening to this one or that one who comes with advice, but it is up to us* (female, 38).

Only one 29-year-old female participant reported feeling anger towards her partner when she learned his HIV diagnosis. She reported feeling betrayed because her partner’s diagnosis exposed his continued extra-marital affairs. She was irritated that, ‘his health situation … forced me to take these drugs’ (female, 29). Despite these feelings, she stayed with her partner and continued to take PrEP for more than a year.

#### Knowledge about PrEP and the belief it is effective

Participants reported low levels of knowledge about PrEP before receiving a discordant diagnosis. The limited efforts to educate community members about PrEP, which has led to low demand for the medication (a substantial barrier to uptake), have also decreased misconceptions or conspiracy theories like those that surround HIV medication. Nurse-led education about and access to PrEP enabled couples to feel comfortable with their decision to stay together. One participant elaborated, ‘I didn’t even know that there was a PrEP treatment so for both of us it was a great victory to know that he will be much better, and I will be protected from the HIV virus’ (female, 37). One woman even expressed relief at finally having a diagnosis,

*… we were already tired going to several great people (traditional medicine practitioners), but it didn’t get better, and it got worse. That was when he decided to come to the hospital to do the test. Everyone is taking their medication and we are fine* (female, 25).

#### Fear of HIV and PrEP stigma

While PrEP itself was not well-known, HIV remained highly stigmatized. Many participants feared stigma if other members of their family or friends were to find out that their partner was HIV-positive or that they were taking PrEP. One participant reported, ‘ … in the community people react in strange ways to people who are living with HIV … That’s why we don’t tell anyone about our situation’ (male, 32). Few disclosed their status to more than one or two family members.

A few participants expressed their anticipated stigma as a fear of witchcraft or curses from community members. One woman commented, ‘if people know about your condition, they can increase your disease with a spell to make people think it is HIV’ (female, 38). Another woman commented that, ‘[If I say] that my husband has HIV, other people can take advantage and cast witchcraft on us’ (female, 26).

### Interpersonal factors affecting PrEP uptake

#### Desire to protect family

Both men and women spoke about the importance of adherence, particularly to ensure the wellbeing of their children. One man noted that his ‘concern at the moment was if I was taking these drugs myself, I would not get the disease that my wife has, and our children end up suffering from the disease that both parents have’ (male, 32). Although almost all participants reported feeling sad about their partner’s HIV diagnosis, they were not angry, nor did they threaten to leave. One participant explained,

*I had this thought: she didn’t want it [HIV], I didn’t want it either. It is God’s destiny. If I had the idea of leaving her, I can find something worse out there, so I preferred to stay with her* (male, 35).

#### Partner support and relationship strength

Most participants reported working with their partner to improve ART and PrEP uptake and adherence. Few believed that someone could manage to take PrEP without the support of their partner. One, in response to a story designed to elicit an opinion about a woman using PrEP without her partner’s knowledge, said,

*I don’t think she will make it. She will take it for a while, but then she will give up because it will not be possible for both of them in the same house … to keep this secret for a long time. I think this woman will not even reach a year to take the medication because they are in disagreement* (male, 39).

Participants afraid to disclose their discordant relationship status and use of PrEP agreed that a person’s health status was between them and their partner. The main issue that undermined disclosure was HIV stigma, specifically anticipated stigma. While couples appeared to be able to navigate a positive test result with their partner, they did not believe that others would be as kind towards the HIV-positive partner’s status. One reported,

*… many people are discriminated against for that disease [HIV] he [my husband] does not want anyone to know, because that family will also tell someone else, someone else will tell another, it will no longer be a secret to anyone* (female, 29).

#### Overcoming the fear of stigma to seek support from family and friends

Participants who had not disclosed their discordant relationship status and use of PrEP to family and friends were concerned about how others would talk about and act towards them or their partner. One participant explained, ‘here in the field, when they know that the couple undergoes treatment … they just talk about it, so even if you were active [feeling well] you will start to be weak and demoralized’ (male, 39). Another agreed, describing how if, ‘someone also starts to know about it [our situation] you are very humiliated’ (female, 29).

Most participants who reported sharing their situation with family or friend(s) reported that it was a positive experience. Some participants told one family member or friend but were too afraid to tell others. One woman reported that her grandmother, ‘liked it [PrEP] because her granddaughter doesn’t have the disease [HIV]’ (female, 18). Others noted that family members stressed the importance of continuing to take PrEP, like one man, whose brother told him, ‘you cannot give up’ (male, 27). One man even reported that when his partner stopped taking her ART, her ‘sister came and counselled her and explained to her what the paths are and from there she started on her [antiretroviral] course till today’ (male, 38). When asked if they would recommend that others seek support from friends or family, one woman explained:

*I was going to call only his closest family, parents, and siblings, because sometimes someone denies treatment not because they don’t want to, but because they are frustrated with the disease and need someone’s strength or advice* (female, 38).

#### Gendered adherence approaches

There were differences in how female and male partners approached HIV treatment in their seropositive partner. Female partners reported pressuring their male partners to start PrEP and adhere to treatment, while male partners framed adherence as collaborative. Female participants tended to see themselves as the dominant force in health care seeking and treatment adherence. One reported that she, ‘tend[s] to force him [her partner] to take the medication … until I have the proof that he already took it, and he should also make me’ (female, 29). Another reported,

*I was the one who forced my husband to come to the hospital for a test. He didn’t want [to] and I didn’t even know that there was a PrEP treatment so for both of us it was a great victory to know that he will be much better and I will be protected from the HIV virus* (female, 37).

One man, aware of this dynamic, reported, ‘[My wife] told me to always get up and not stop taking [PrEP]’ while simultaneously expressing the solidarity he and his partner found in staying adherent to their medicines, ‘for her to live, and for me to live too!’ (male, 39). Another man said that it was his ‘wife who gave [him] the most strength’ (male, 22) and a third reported, ‘the main strength we have is that after dinner we both take [PrEP and ART together]’ (male, 35). This description of solidarity reflected the belief males shared that to succeed in their treatment, the couple must work together for the benefit of the family.

### Organizational/clinic factors affecting PrEP uptake

#### Welcoming clinicians

Most participants spoke in positive terms about their interaction with health care workers, counsellors, and peer activists. Almost all participants were satisfied with the system in place to collect their medications at the health facility. They found it comforting to hear medical information from medical professionals; and they felt safe asking questions about PrEP and their partner’s HIV diagnosis at the health facility. One woman explained that the clinic room was ‘safe, they serve us well and we are respected’ (female, 38).

#### Supportive community health workers

Participants also highlighted the importance of support from community health workers. One reported, ‘Those activists who pass in the communities, help [me] understand some things … congratulated me … and gave me a lot of strength’ (male, 39). A woman described her positive experience with nurses who visited community members in their homes to provide information and answer their questions. Another woman concurred, explaining in response to a question about the best way to support serodiscordant couples,

*There are those people in the neighborhoods giving advice to couples, they may be able to advise them to get along and do the treatment. Because those who pass by the houses to give advice have helped many couples … They know the tactics for talking to such [difficult] people and are very patient people* (female, 32).

### The creation of oral stories to help future discordant couples

The above responses guided the template for the three stories aimed to increase PrEP uptake and adherence in Zambézia province (Table 2). The first story is designed to empower the couple to remain in care and adhere to treatment by presenting the experience of a couple who successfully start and stay to their medication. The second story provides strategies to overcome stigma and anger from the couple’s extended family. The third story focuses on strategies to manage an unsupportive spouse, including support that is available at the health facility and within the community. Each story presents potential barriers to PrEP adherence from various levels of the socioecological model, and examples of how couples can use resources available at different levels to overcome those barriers.

## Discussion

There are more than 30 studies assessing the impact of biobehavioral interventions among serodiscordant couples in sub-Saharan Africa [30]. These studies were designed to increase knowledge and develop psycho-educational skills [30–32], encourage couples-based HIV testing and counseling [33,34], and promote PrEP and ART uptake [35–37]. Across these studies, male partner attitudes and behaviors have a significant impact on uptake of, and adherence to, medication among women [38–40]. Similarly, our participants indicated how essential their partner’s support was for their PrEP (and ART) adherence. Although most participants felt comfortable staying in a serodiscordant relationship, women taking PrEP felt that they needed to pressure their partner to adhere to his ART, whereas men taking PrEP saw themselves engaged in collaborative relationships that promoted medication adherence. Similar experiences have been reported by women in seroconcordant, HIV-positive, relationships [41]. Harnessing the power of the couple to support adherence to medication may therefore require different strategies directed towards women and men. For example, men can often exert more control of their partner’s ART adherence than women, making PrEP a lifeline for women with partners with poor adherence [40]. Interventions that address stigma would be more effective if directed towards women who report greater fear of disapproval from partners or the community [42–44]. Although, different approaches will need to be cognizant of gender-based power imbalances that may, at their extreme, manifest as interpersonal violence and decreased PrEP adherence among female partners [45].

There also appears to be a disconnect between the actual and presumed attitudes towards HIV and PrEP among people in the community. Despite positive experiences among those who disclosed their status, participants reported a strong fear of stigmatization or discrimination from their community, which is consistent with other studies [46–48]. Previous research has also found stigma associated with PrEP-use disclosure and PrEP’s association with HIV [49–51]. Our participants’ fear of stigma was generally focused on how their community viewed their partner’s HIV status, not their use of PrEP. This presents an opportunity to shape how PrEP is perceived through coordinated implementation strategies, such as our oral stories, that directly address anticipated stigma among people living with HIV and within communities. Developing and delivering engaging stories has proved successful for several reasons: (1) Stories act as mnemonic devices for facts [52,53]; (2) Stories engage emotions, resulting in increased empathy and adoption of messages [54]; and (3) Stories that engage participants lead to story-consistent beliefs [55]. Given the history of oral storytelling as education in Mozambique, this culturally-informed tradition can be used to reduce HIV stigma and increase understanding of complex topics like PrEP and ART use [27–29].

Finally, interviewed couples suggested expanding the use of HIV community health agents to PrEP treatment support. Peer supporter programs, like similar ART programs, could result in decreased internalized stigma as well as increased knowledge and confidence in a client’s ability to take their medication regularly [16,17,56]. This strategy could help serodiscordant partners make well-informed decisions for themselves and their families in similar communities and further normalize HIV and intra-couple discordance, which would improve PrEP uptake and decrease PrEP-related stigma and HIV transmission. Community health agent involvement can also expand the use of storytelling. Given low education levels in most rural areas in Mozambique, telling stories engages those taking medication and, if the couple is willing, their family. Given the importance of social support for long-term treatment adherence [57–59], family engagement in listening and responding to stories about ART and PrEP use among couples can provide an additional level of support for discordant couples.

### Methodologic considerations

The results of this qualitative study are from one study site in rural Mozambique and may not transfer to perspectives in urban regions of Mozambique where PrEP knowledge is higher and attitudes towards PrEP may have already been established. Except for three participants, we did not capture the opinions of people who initiated PrEP but discontinued medication or those who were eligible but decided not to enroll in treatment. The general themes from this study, however, which align with other research in the field and were consistent among participants, will allow us to develop, test, replicate, and expand interventions to increase PrEP more broadly in rural Mozambique. Once we develop messaging and study its implementation among other community members, we will be in a better position to explore why some serodiscordant couples are not using PrEP, which we can then integrate into future interventions aimed at increasing PrEP uptake.

### Next steps

We have integrated participant responses into three oral stories that will be delivered to serodiscordant couples, and if acceptable, their family. These stories target the individual, interpersonal, and organization levels of the socioecological model to increase PrEP uptake and reduce HIV transmission. A theatre-trained couple will present these stories to 35 randomly selected discordant couples at treatment initiation, two weeks, and one-month post enrollment. We will compare adherence to PrEP among this intervention group with those in standard of care. This will allow us to assess the efficacy of these stories, make modifications as appropriate, and implement similar story-driven interventions at other sites in rural Mozambique (Clinical Trial NCT04071470).

## Conclusions

Policies aimed at increasing PrEP uptake in communities must engage serodiscordant partners and train community health agents to disseminate comprehensive information beyond clinic walls. These measures will likely allow rural health systems to increase PrEP uptake and reduce HIV transmission. Future studies should assess PrEP messaging and determine how it impacts PrEP uptake in different communities, given the need to reduce HIV transmission across Mozambique and beyond.

## Paper context

Pre-Exposure Prophylaxis (PrEP) is an essential component in stemming the HIV epidemic globally, however, PrEP uptake in sub-Saharan Africa is suboptimal. This paper elicits individuals in serodiscordant partnerships’ opinions about facilitators of and barriers to PrEP use in rural Mozambique. Their answers have helped guide educational materials for clinics in the region, which will be studied further prior to implementation in other regions in Mozambique.
